# The Transantral Endoscopic Approach: A Portal for Masses of the Inferior Orbit—Improving Surgeons' Experience Through Virtual Endoscopy and Augmented Reality

**DOI:** 10.3389/fsurg.2021.715262

**Published:** 2021-08-23

**Authors:** Alessandro Tel, Lorenzo Arboit, Salvatore Sembronio, Fabio Costa, Riccardo Nocini, Massimo Robiony

**Affiliations:** ^1^Department of Maxillofacial Surgery, University Hospital of Udine, Udine, Italy; ^2^Faculty of Medicine and Surgery, Sant'Anna School of Advanced Studies, Pisa, Italy; ^3^Department of Otorhinolaryngology, University Hospital of Verona, Verona, Italy

**Keywords:** endoscopic surgery, virtual surgical planning, virtual endoscopy, navigation, augmented reality

## Abstract

In the past years, endoscopic techniques have raised an increasing interest to perform minimally invasive accesses to the orbit, resulting in excellent clinical outcomes with inferior morbidities and complication rates. Among endoscopic approaches, the transantral endoscopic approach allows us to create a portal to the orbital floor, representing the most straightforward access to lesions located in the inferior orbital space. However, if endoscopic surgery provides enhanced magnified vision of the anatomy in a bloodless field, then it has several impairments compared with classic open surgery, owing to restricted operative spaces. Virtual surgical planning and anatomical computer-generated models have proved to be of great importance to plan endoscopic surgical approaches, and their role can be widened with the integration of surgical navigation, virtual endoscopy simulation, and augmented reality (AR). This study focuses on the strict conjugation between the technologies that allow the virtualization of surgery in an entirely digital environment, which can be transferred to the patient using intraoperative navigation or to a printed model using AR for pre-surgical analysis. Therefore, the interaction between different software packages and platforms offers a highly predictive preview of the surgical scenario, contributing to increasing orientation, awareness, and effectiveness of maneuvers performed under endoscopic guidance, which can be checked at any time using surgical navigation. In this paper, the authors explore the transantral approach for the excision of masses of the inferior orbital compartment through modern technology. The authors apply this technique for masses located in the inferior orbit and share their clinical results, describing why technological innovation, and, in particular, computer planning, virtual endoscopy, navigation, and AR can contribute to empowering minimally invasive orbital surgery, at the same time offering a valuable and indispensable tool for pre-surgical analysis and training.

## Introduction

Technological development represented a powerful impulse in the way surgeons changed their attitude toward surgical approaches. In orbital surgery, this meant moving from traditional transcutaneous incisions to an increasing application of minimally invasive techniques in order to excise pathological masses. For instance, orbital endoscopy has surged in popularity in the past years, due to its limited invasiveness and magnified visualization (1–3).

In addition to endoscopy, modern maxillofacial surgery continues to benefit from technological improvement, which has led to a deep change in the conceptual approach to the pre-operative study of the patient, including simulation and training for individual cases.

Modern medical software has the power to create entirely virtual environments with a high degree of correspondence to reality, including the possibility to perform accurate modeling of structures using multiple imaging techniques for both bone and soft tissues. Moreover, design and animation software can replicate complex combined movements of objects, including deformations, and provide the user with the possibility to place multiple cameras, which can be seen through and animated following a pre-defined path, allowing to simulate a fully endoscopic view for each surgical maneuver (4).

Evolution of 3D printing has brought to the clinician the possibility to manufacture trustful replicas of virtual objects (5), while surgical navigation allows us to track step-by-step the position of surgical instruments in the operating field (6, 7).

Recent developments in the field of augmented reality (AR) have provided powerful software engines for object recognition and motion tracking, enabling to bring on mobile devices the possibility to superimpose virtual entities on the real-world targets (8).

A prominent example of this evolution is represented by orbital lesions arising in the inferior orbital space, which continue to be treated conventionally. Anatomically, the transmaxillary corridor has the peculiarity to provide the most straightforward access to the orbital floor, thus allowing the most direct vision of the inferior orbit (9).

In this study, the authors present how the meticulous use of technology allows us to take advantage of the surgical benefits of the transantral corridor to excise masses arising in depth in the inferior orbit. Technology appears in its main declinations in maxillofacial surgery: computerized virtual surgical planning, 3D printing, intraoperative navigation, and AR. The result is the adoption of the transmaxillary approach as the first choice for masses located in the inferior orbital compartment. We describe the workflow employed in our case series, including advantages for pre-operative study and simulation, in a mindful blending of technological resources, which are today available to the modern maxillofacial surgeon.

## Materials and Methods

Five patients were enrolled for this study in a time span ranging from January 2019 to April 2021. Table 1 details their demographic and clinical–pathological features. Patients came to clinical attention complaining of at least one of the following symptoms: eye swelling with globe proptosis in the past months, progressive onset of diplopia, and gradual loss of visual acuity. To be eligible for transmaxillary endoscopic surgery, patients had to fulfill the following inclusion criteria: the presence of an intraorbital mass located over the orbital floor, absence of sinusitis or maxillary sinus hypoplasia, radiological features suggestive for benignancy or, at least, evidence of well-defined boundaries of the lesion. This study was conducted in accordance with the declaration of Helsinki and is part of the protocol IRB_45_2020 approved by the Institutional Review Board (IRB) of the University of Udine.

**Table 1:** Demographic and surgical characteristics of patients enrolled in this study.

**Patient ID**	**Gender**	**Age**	**Surgical time (min)**	**Localization**	**Histopathology**	**Orbital floor reconstruction**	**Complications**
1	M	57	150	Medial to infraorbital canal	Cavernous	Antral wall graft	Transient ION paresthesia
					Hemangioma		
2	M	49	100	Medial to infraorbital canal	Neurofibroma	Original floor	None
3	F	63	210	Medial to infraorbital canal	Cavernous	Antral wall graft	Transient ION paresthesia
					Hemangioma		
4	M	59	130	Lateral to infraorbital canal	Cavernous	Antral wall graft	Transient ION paresthesia
					Hemangioma		
5	F	55	90	Medial to infraorbital canal	Schwannoma	Original floor	None

*ION, infraorbital nerve*.

### Reconstruction of the Virtual Patient

**Figure 1:**
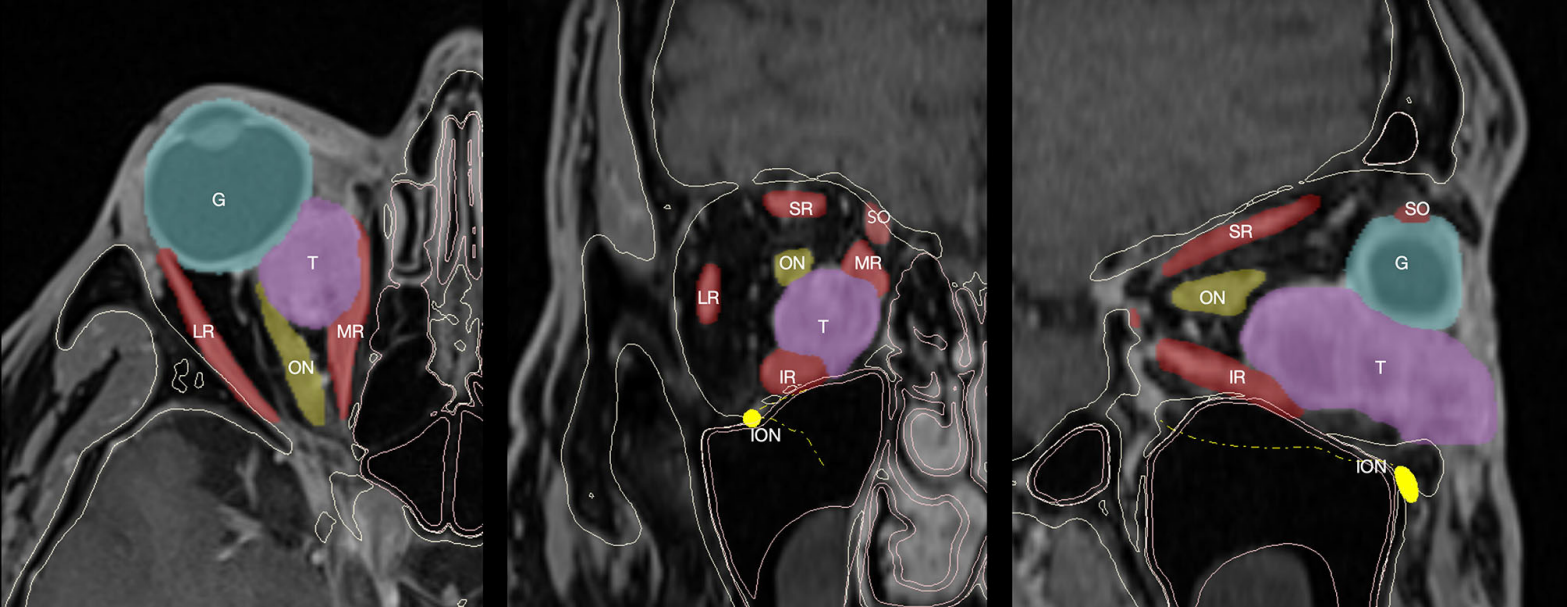
CT and MR coregistration and segmentation. Axial, coronal, and sagittal projections are reported from left to right. Segmentation masks are defined as follows: the eye globe (G), tumor (T), optical nerve (ON) and infraorbital nerve (ION), extraocular muscles, including superior rectus (SR), lateral rectus (LR), inferior rectus (IR), medial rectus (MR), and superior oblique.

**Figure 2:**
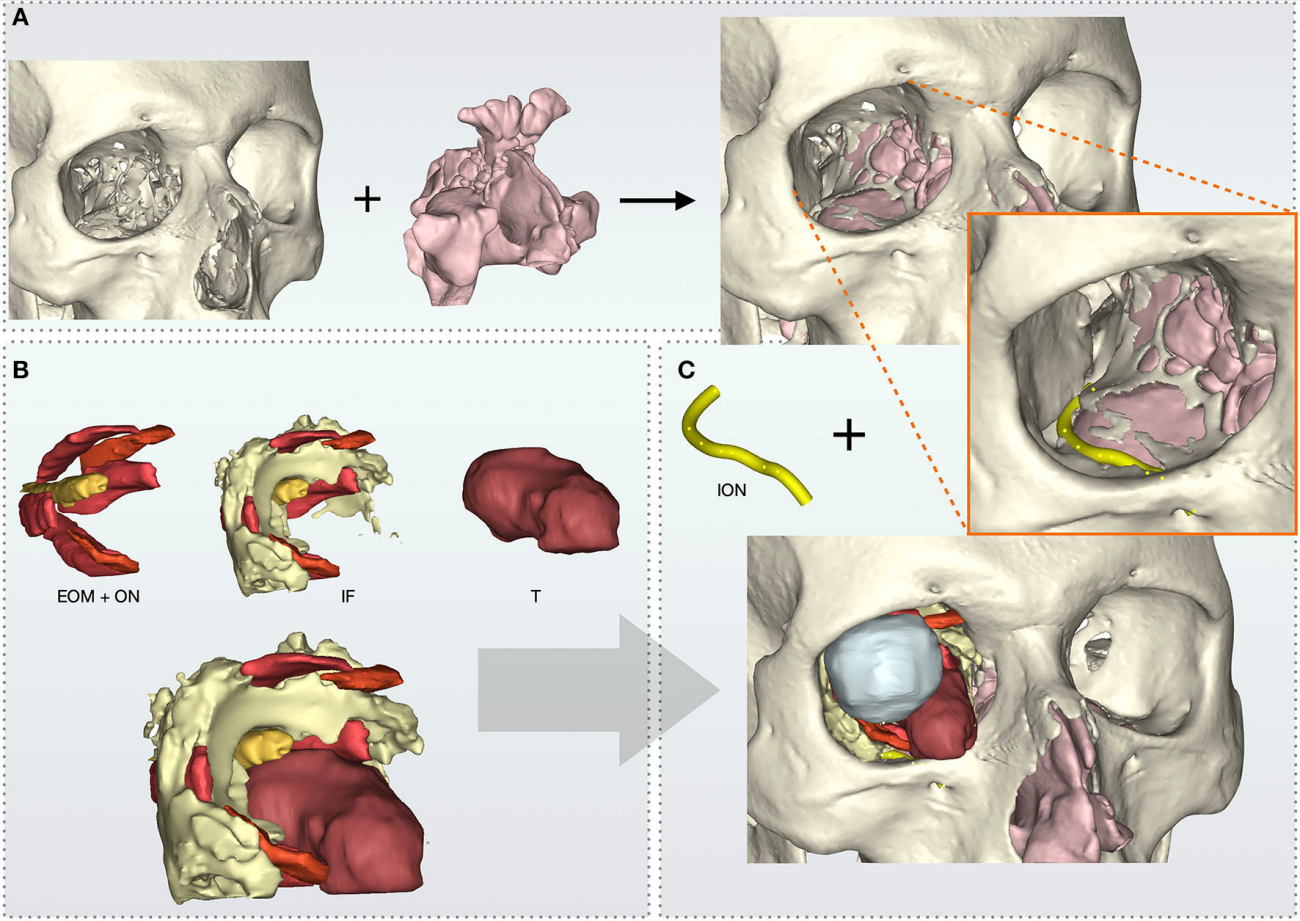
Virtual model reconstruction workflow of intraorbital structures, skull, and paranasal sinuses. **(A)** Paranasal sinuses model is composed with skull. **(B)** Extraocular muscles (EOM) and optical nerve (ON) are grouped with intraorbital fat (IF) and tumor's (T) models. **(C)** Complete anatomical model with the addition of infraorbital nerve (ION).

In order to perform a computerized simulation of surgery, the first step is to replicate the real anatomy in a virtual environment. To acquire bone anatomy, all patients underwent volumetric CT scan with isotropic voxel, a 512 × 512 matrix, and a 0.6 mm slice thickness. Intraorbital lesions were studied using MR with the following parameters: sequences for optimal anatomical visualization, including enhanced VIBE-T1W, 3D-T2, and 3D-T1 with 1 mm slice thickness and isotropic 512 × 512 matrix, eventually resliced into 0.6-mm slices for optimal superimposition with the CT. DICOM data were imported within the software Mimics v23.0 (Materialise, Leuven, BE), where CT and MR were coregistered using an automatic registration function, yielding paired image sets with a shared coordinate system. Within Mimics, bone structures were semi-automatically segmented from the CT scan. The roof and the anterior wall of the maxillary sinus were thoroughly reconstructed to preserve all the bone contour, as this region is very thin, and the partial volume effect oftentimes causes artifactual bone loss on the virtual model. Moreover, thresholding directed to the mucosal lining density (range: 250–800 HU, Hounsfield Units) was used to reconstruct soft tissue paranasal sinuses. Therefore, soft tissue lesions were segmented from MR imaging initially using a combination of semi-automatic methods, including thresholding and dynamic region growing. Segmentation masks were further refined under the assistance of the radiologist applying a mask split function across the boundaries of the lesion and performing slice-by-slice editing on the most critical points. The same process was repeated for each anatomical subunit, to accomplish a detailed anatomical reconstruction of the intraorbital space, including the eye globe, the extraocular muscles, and the optical nerve. The intraorbital fat was obtained by subtraction of the already-segmented structures from the whole intraconal volume (Figure 1). Segmentation masks were then tessellated to be converted into three-dimensional objects and were exported as individual STL (Standard Tessellation Language) files. We suggest reconstructing the models with a high polygonal resolution to preserve intact anatomical detail: In the example shown in Figure 2, the tumor consists of ~30.000 triangles with 15.000 points. Particular care was taken to reconstruct the infraorbital nerve that is crucial when planning a transantral approach through the orbital floor. Once the reconstruction of anatomical models is complete, it is possible to explore the virtual orbit in every dimension, hide and show selectively any part, and draw osteotomies. Moreover, geometrical parts represent the pre-requisite to recreate a scene setup suitable for the digital animation and the simulation of virtual endoscopy.

### Virtual Surgical Planning

**Figure 3:**
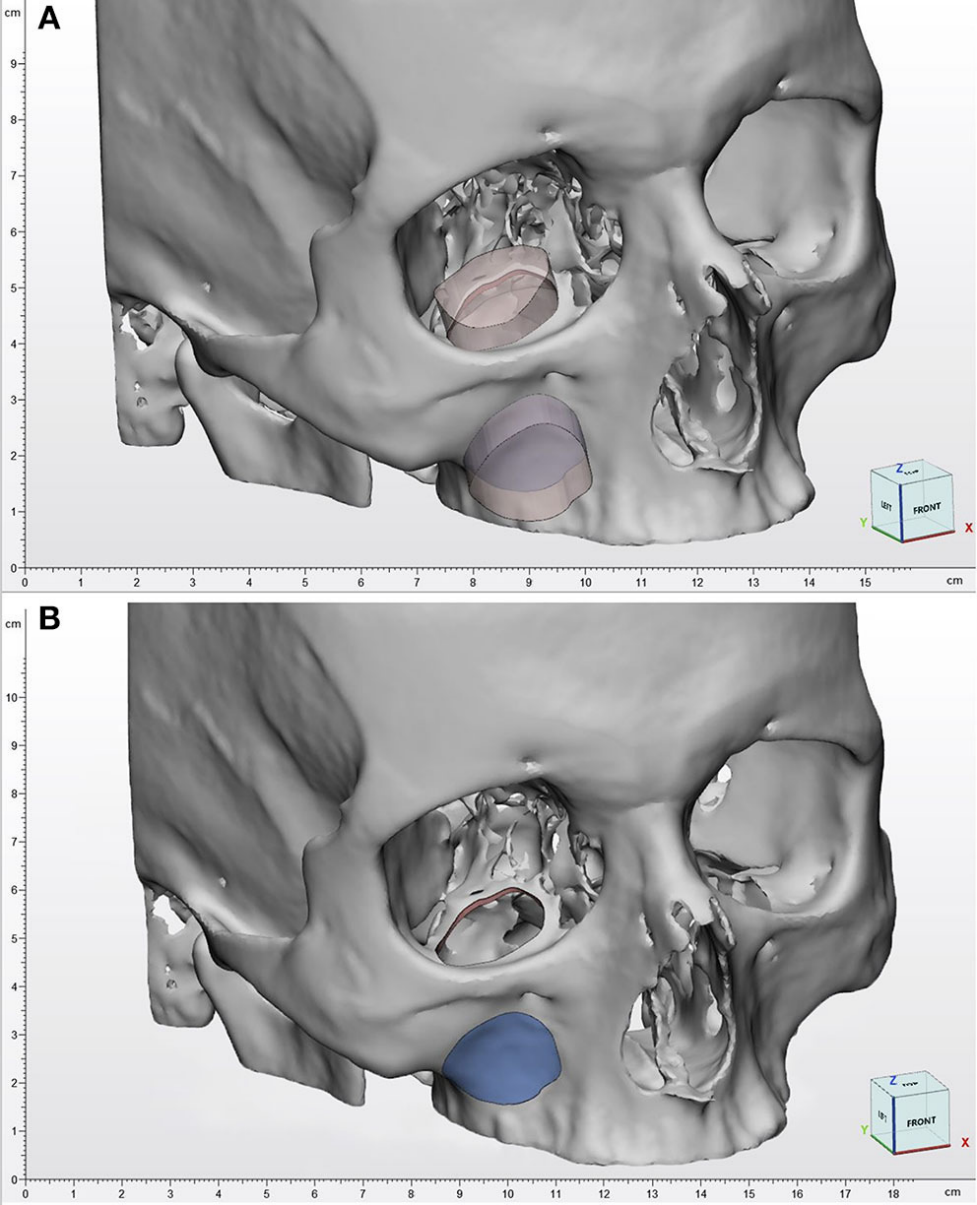
Virtual surgical planning. **(A)** The transmaxillary and the transorbital portal are defined from a single geometrical template and are therefore correlated. **(B)** As a consequence, the orbital osteotomy template is used as a reference to design the maxillary wall osteotomy, to facilitate orbital floor reconstruction using an antral wall graft.

The STLs of anatomical parts were imported in 3-Matic software (Materialise, Leuven, BE), where virtual surgical planning was performed to simulate a transantral access. Two interrelated elements are to be defined in the virtual plan of a transantral access to orbital masses located over the orbital floor: the transmaxillary portal and the transorbital portal (Figure 3A). First, the lesion was intersected with the orbital floor to determine the optimal size of the osteotomy, which could enable the lesion to smoothly pass through the orbital floor. The orbital floor was pierced accordingly, and the resulting hole was taken as a reference to design the maxillary wall osteotomy using the same subtraction template (Figure 3B). It is important that the maxillary wall osteotomy be wide enough for the comfortable passage of the optics and surgical instruments within the sinus; moreover, the excised bone dowel might be used to reconstruct the orbital floor if the lesion has caused a bone erosion or if the bone breaks up when the osteotomy is performed. Once planning is complete, modified STL files are used to recreate the virtual scene in the animation software; moreover, they can be imported into the software iPlan CMF 3.0 (Brainlab, Munich, Germany) to create a navigation plan for transmaxillary navigation. Each skull and its surgical guide for the transmaxillary portal were 3D-printed to be used for AR tracking (the skull) and maxillary wall osteotomy (the guide).

### Animating the Sequence for Virtual Endoscopy

**Figure 4:**
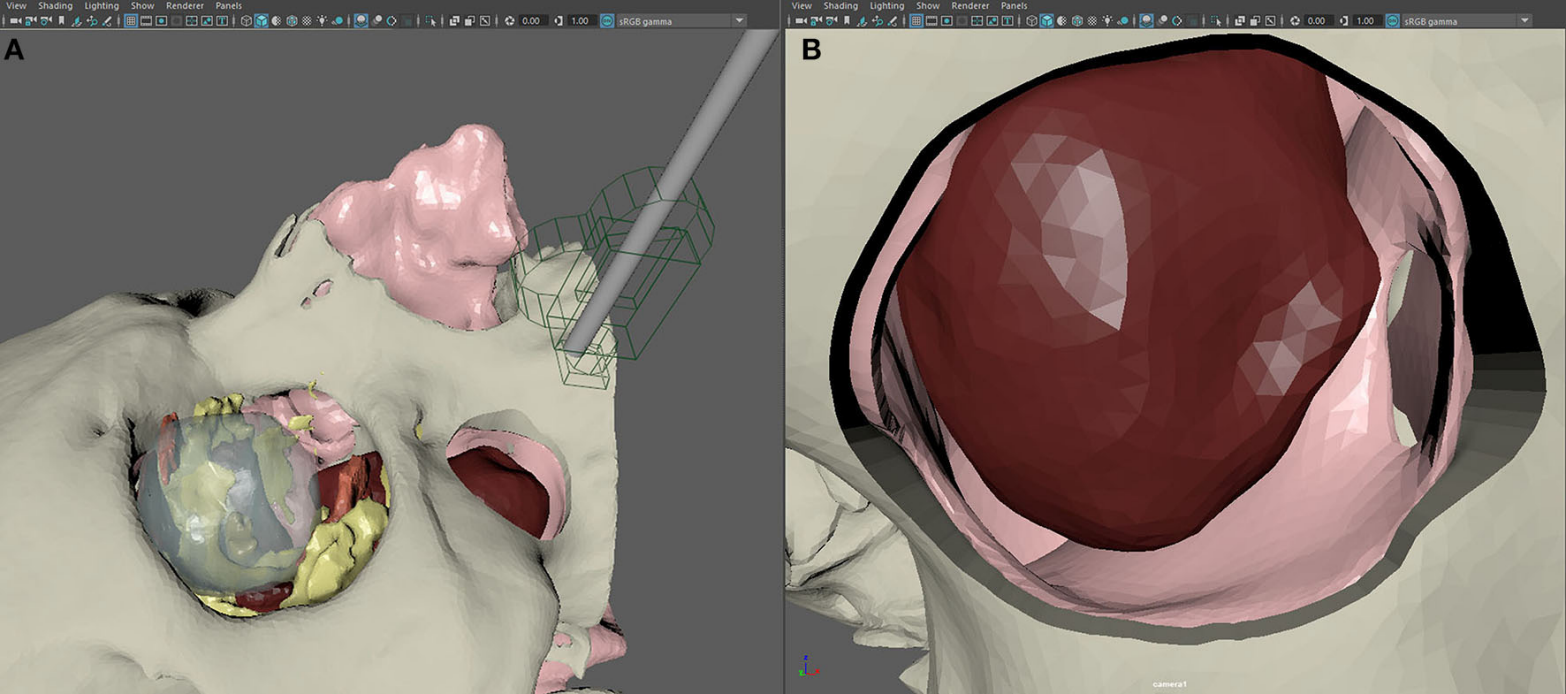
Setup of the scene in Autodesk Maya for the creation of virtual endoscopy. **(A)** The endoscope is recreated in its real size using a geometrical replica and a virtual camera. **(B)** Simulated endoscopic view of the transmaxillary portal, the paranasal sinuses, and the tumor.

Individual STL files were imported in the software Autodesk Maya (Autodesk Inc., San Jose, CA, United States), a powerful 3D package that represents the industry standard for complex 3D animations. Maya requires to set up a “scene” project, namely, a virtual environment where the user defines not only the position of single geometrical entities, but also lights and cameras, although it provides default cameras for orthographic projections and perspective visualization, as well as a default lighting system. For the specific aim of simulating a fully endoscopic procedure, we reproduced a scene similar to the intraoperative scenario, in which the head of the patient is tilted slightly backward to allow easy optics insertion. Optics was simulated using a cylinder of the same size and caliber with a camera on its extremity, whose angulation was regulated to recreate a 0, 30, and 45° optics. The optics see-through function allowed us to inspect the virtual maxillary antrum exactly as if the surgeon were using a real endoscope. The virtual camera, mimicking the endoscope, was configured following the parameters provided by the manufacturer (KARL STORZ SE & Co. KG, Tuttlingen, Germany) using an image refreshing of 50 fps and a focal distance of 18 mm (Figure 4).

The first step in animation was to represent the removal of the anterior wall of the maxillary sinus according to the virtual plan, which is necessary to introduce the optics within the maxillary antrum to perform a transantral approach to the orbital floor. Second, the orbital floor osteotomy was simulated from within the maxillary sinus: As this procedure is conducted entirely endoscopically, the simulation had the aim to recreate a foreseeable situation. The third step was to animate the tumor removal: Using three-dimensional manipulators to move objects in the virtual scene, we simulated the passage of the tumor through the orbital floor osteotomy and its removal through the opening designed on the anterior wall of the maxillary sinus. A lattice deformer was applied to the inferior rectus muscle and the infraorbital nerve to simulate their deformation when displaced by the traction maneuvers or the passage of the bulky tumor mass. The fourth step was represented by the animation of the orbital floor reconstruction, using the originally excised orbital floor or, if damaged by the osteotomy, the anterior wall of the maxillary sinus as well. The last animation step was dedicated to the camera: From the camera see-through panel, the operator moved the camera by setting its position in each key frame across the timeline, allowing the software to register the positional variation of the camera over time in the correct sequence for the surgical maneuvers. The whole animated sequence is shown in Supplementary Video 1.

Animation was refined and made more fluent by selectively smoothing and adapting movements of geometries and camera using the graph editor in Maya, which enables complete control over animation curves of single movements. The animation was rendered as a whole video sequence. The scene was exported from Maya in the FBX (FilmBoX) format, generating a single file containing the whole scene, including individual objects and their animation. Supplementary Video 1 shows the full animated sequence.

### Introducing AR Pre-surgical Simulation

Unity (Unity Technologies, San Francisco, United States) and Vuforia Engine (PTC, Boston, United States) were used to recreate an AR environment where we simulated endoscopic procedures on the patient-specific printed model. First, Vuforia Engine SDK (software development kit) was added to the Unity project, allowing us to integrate AR features into the Unity cross-platform game engine. Vuforia license key was set as default through the License Manager in the developer portal; from the same portal, we exported a Target Manager database with a custom image that was later used as a locator for the virtual screen where the endoscopic view was simulated. Skull STL model was imported into Model Target Generator (PTC, Boston, United States) application: it provided a Vuforia Database which allows the Vuforia Engine to track the corresponding real-world object; optimal tracking was achieved with Advanced Views set at 360° on the transverse plane and 180° on the sagittal plane.

**Figure 5:**
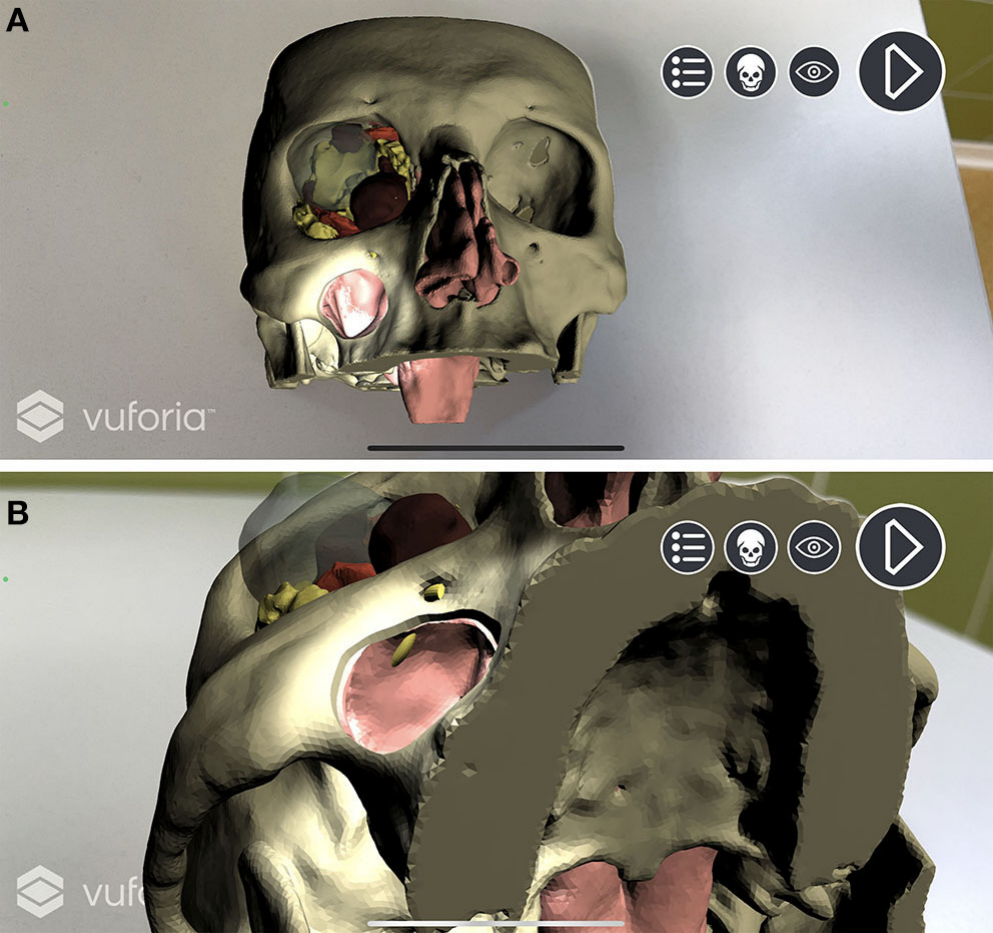
Augmented reality simulation. All components of the virtual model are rendered over the physical 3D printed model. **(A)** Frontal view with simulated lightning over the transmaxillary portal. **(B)** Inferior view of the mucosal sinus roof with the infraorbital nerve highlighted in yellow.

In Unity environment, AR camera, model target, image target, and default directional light were set as main objects. FBX data obtained from Maya were imported and unpacked to modify their components. The animation FBX file was optimally rescaled and rotated to match the model target dimensions (scale value: 0.1; rotation value: 90° on the x-axis). Animator controller was set to control the imported animation. The endoscopic lighting was obtained with a spotlight attached to the endoscopic camera (Figure 5).

**Figure 6:**
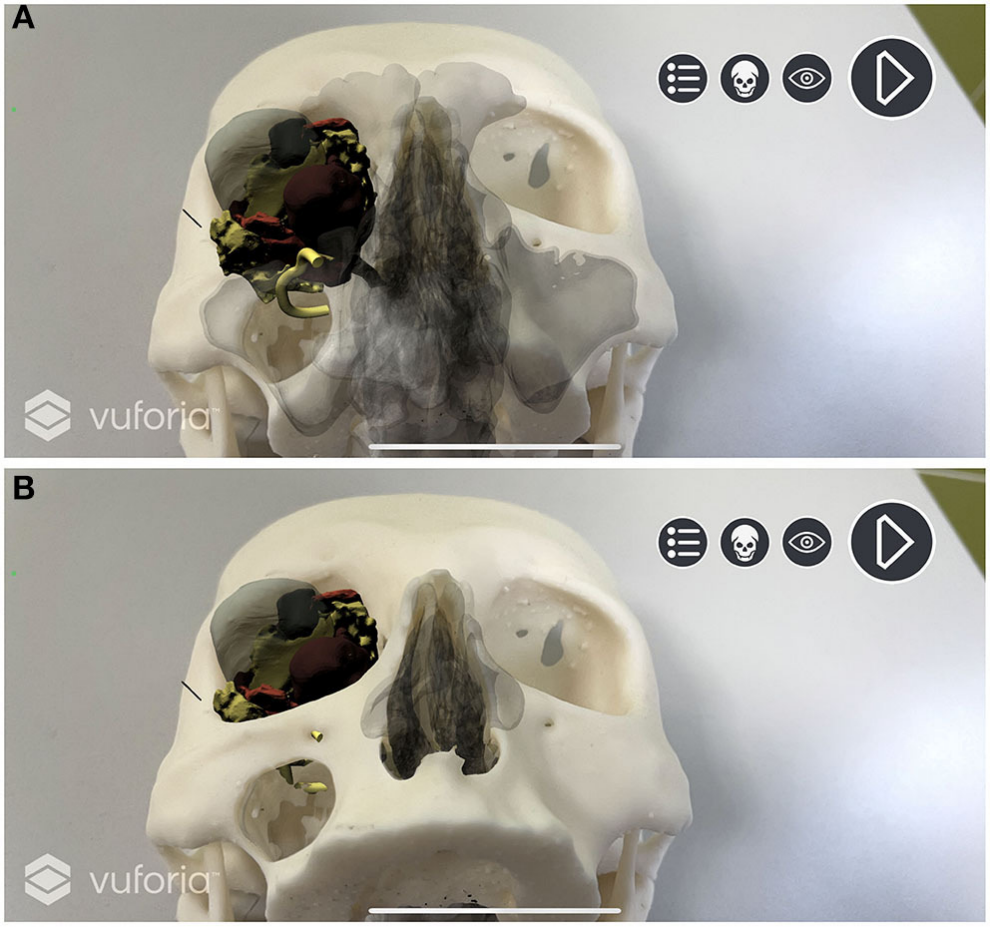
Depth mask rendering. Soft tissues are correctly displayed without the skull virtual model. **(A)** Depth mask is disabled and anatomical structures are simulated as “seen through” the printed model. **(B)** Depth mask is enabled and soft tissues are seen in their natural position on the printed model.

A custom script was programmed to create a standard surface shader, which was applied to the skull STL file to generate a depth mask: This feature superimposes existing objects within the scene, but which are invisible through the camera, in order that they act as a mask. This allows them to still appear in the depth rendering, thus hiding everything that lies behind them. In our application, the virtual skull is therefore rendered as invisible, and the soft tissues are correctly seen in their natural position on the printed model only outside the contours of the skull (Figure 6).

The application was completed with the introduction of a Camera Focus Controller script and a canvas with buttons to reproduce the endoscopic animation and to hide/show every object imported with the FBX file. The project was finally built as an iOS application, and it was run with XCode (Apple Inc, Cupertino, CA, United States) to be tested using an iPhone 12 Pro. A dynamic application of AR simulating the procedure is shown in Supplementary Video 2.

### Navigation-Assisted Surgery

The surgical navigator uses three spatial coordinates to define the position of a rigid body in the space. Therefore, it requires a stereocamera to be paired with a reference frame consisting of a metal tripod with photoreflective spheres was mounted on the head of the patient. Once the patient is visible in the stereocamera field, calibration is performed by univocally associating the real-time position of the probe with the correct location of the virtual patient.

**Figure 7:**
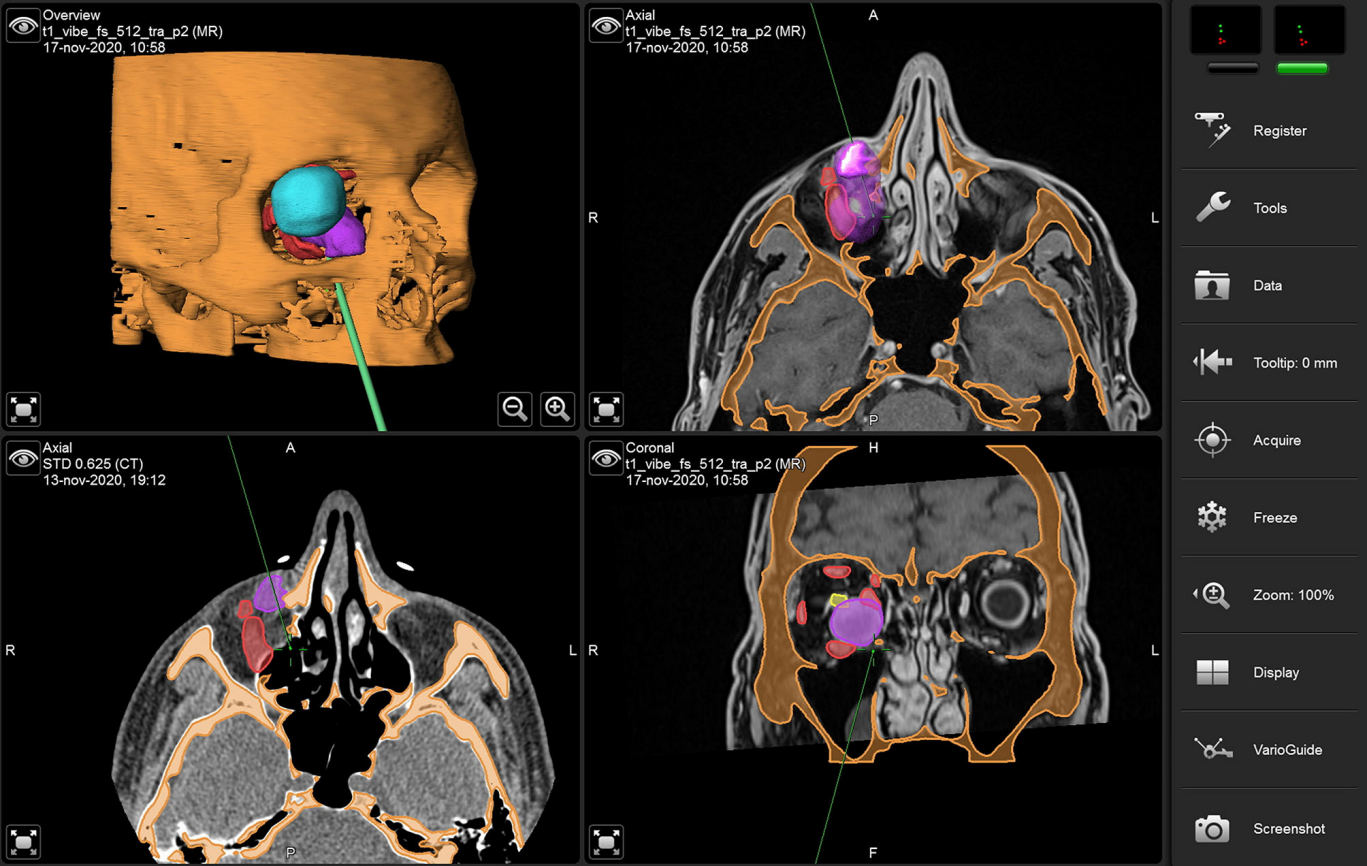
Transmaxillary navigation during surgery using virtual models. The navigation probe indicates the medial edge of the orbital floor osteotomy.

A vestibular incision was performed to gain full exposure to the anterior wall of the maxillary sinus. Periosteum was lifted and a surgical guide was positioned over the anterior maxillary wall to design a bone window sized as the virtual plan. A 0° optics linked to the endoscope was introduced within the maxillary sinus to inspect the orbital floor. A mucosectomy was performed using monopolar cautery, and the orbital floor was exposed with a Freer elevator. An angulated piezosurgical insert was used to conduct the osteotomy of the orbital floor, which was designed under transmaxillary navigational guidance using the virtual template as a reference. Transmaxillary navigation was used as well to check the correct surgical position across all intraoperative phases (Figure 7).

During orbital floor disassembly, the infraorbital nerve was carefully dissected and isolated. Incision of periorbita was performed to access the intraorbital space. Inferior rectus muscle was dissected and isolated and laterally displaced to allow for tumor exposure. Tumor capsule was identified and dissected from intraconal fat, and the mass was progressively grasped using a Weil-Blakesley forceps, until satisfactory mobilization was achieved from the surrounding tissue. As dissection proceeded more distally, transmaxillary navigation allowed for the prompt identification of prominent anatomical landmarks, including the optic nerve. The mass was tractioned through the orbital floor opening (the transorbital portal) and extracted from the anterior maxillary sinus opening (the transmaxillary portal). Orbital floor was reassembled, when possible, using the original bone. However, due to the extreme fragility of this structure, it can oftentimes fracture when it is disassembled; therefore, the anterior wall of the maxillary sinus, grafted with the same size, was used in these cases. Supplementary Video 3 shows the key steps of surgery using the endoscopic transantral approach.

## Results

For all patients, the inferior orbital mass was excised through the transantral access and did not require any external incision. No surgery had to be converted into open approaches. After surgery, three patients reported mild transient intraorbital nerve paresthesia, which disappeared at long time follow-up. No permanent sensory loss was reported. No post-operative retrobulbar hematoma occurred, and no persistent diplopia was reported. Concerning orbital floor reconstruction, only in two patients, the orbital floor was entirely removed and was suitable for reconstruction, while in the other three patients, it broke into several pieces; therefore, the bone dowel from the transmaxillary portal had to be used.

Histopathological examination findings were cavernous hemangioma (three patients); schwannoma (one patient), and neurofibroma (one patient).

**Figure 8:**
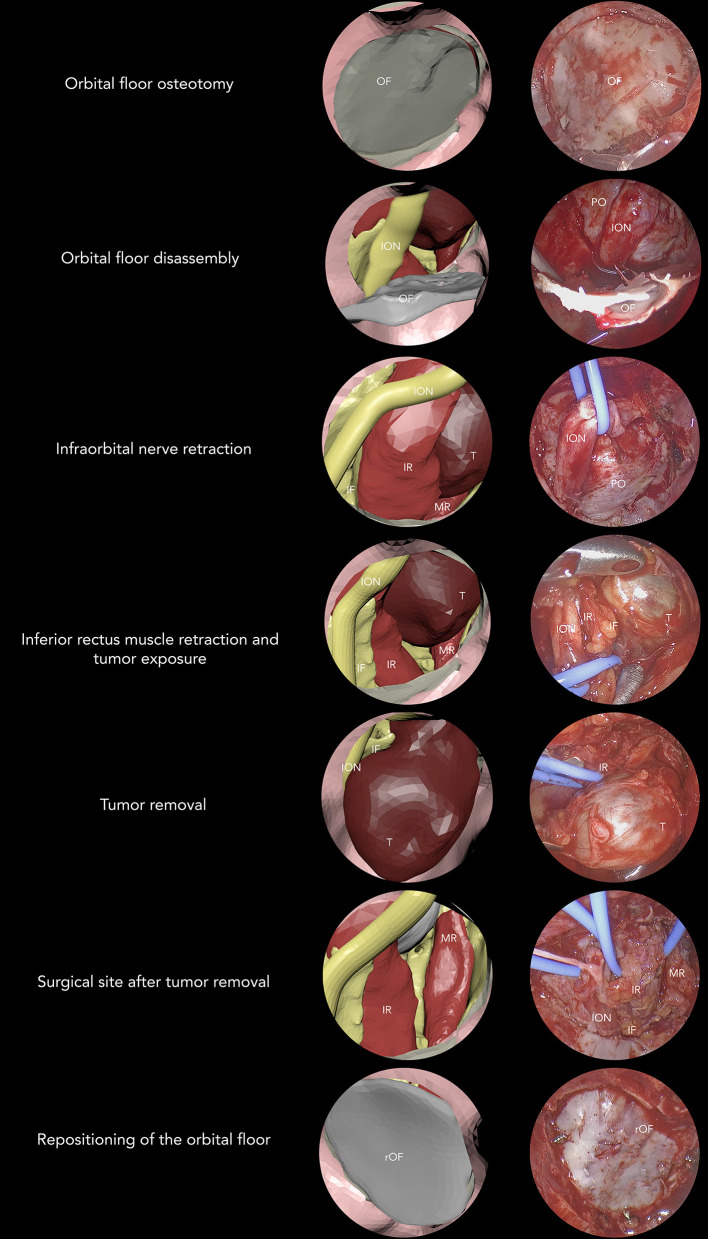
Seven-phase procedure is shown by associating virtual and real endoscopic images. OF, orbital floor; ION, infraorbital nerve; PO, periorbita; IR, inferior rectus; MR, medial rectus; T, tumor; IF, intraconal fat; rOF, reconstructed orbital floor.

To assess whether virtual endoscopic animated sequence could trustfully reproduce the surgical procedure, we compared different surgical sequences with their virtual equivalents, subdividing the endoscopic procedure into seven phases: orbital floor osteotomy, orbital floor disassembly, infraorbital nerve retraction, inferior rectus muscle retraction (with possible tumor exposure), tumor removal, an inspection of the surgical site after tumor removal, reconstruction of the orbital floor (using originally disassembled floor or the antral wall graft). All phases were reproduced in advance in the animated planning and were compared with surgeries for each patient (Figure 8), showing that animated virtual planning could closely reproduce surgical maneuvers. Animated virtual surgical planning was successfully exported as a FBX file, and all objects and their planned movements were preserved exactly as planned in Maya.

Concerning AR implementation, all 3D printed replicas were efficiently tracked, and computer-generated visual information was superimposed for each patient in the correct position. For each case, a separate iOS application was built and stored.

**Figure 9:**
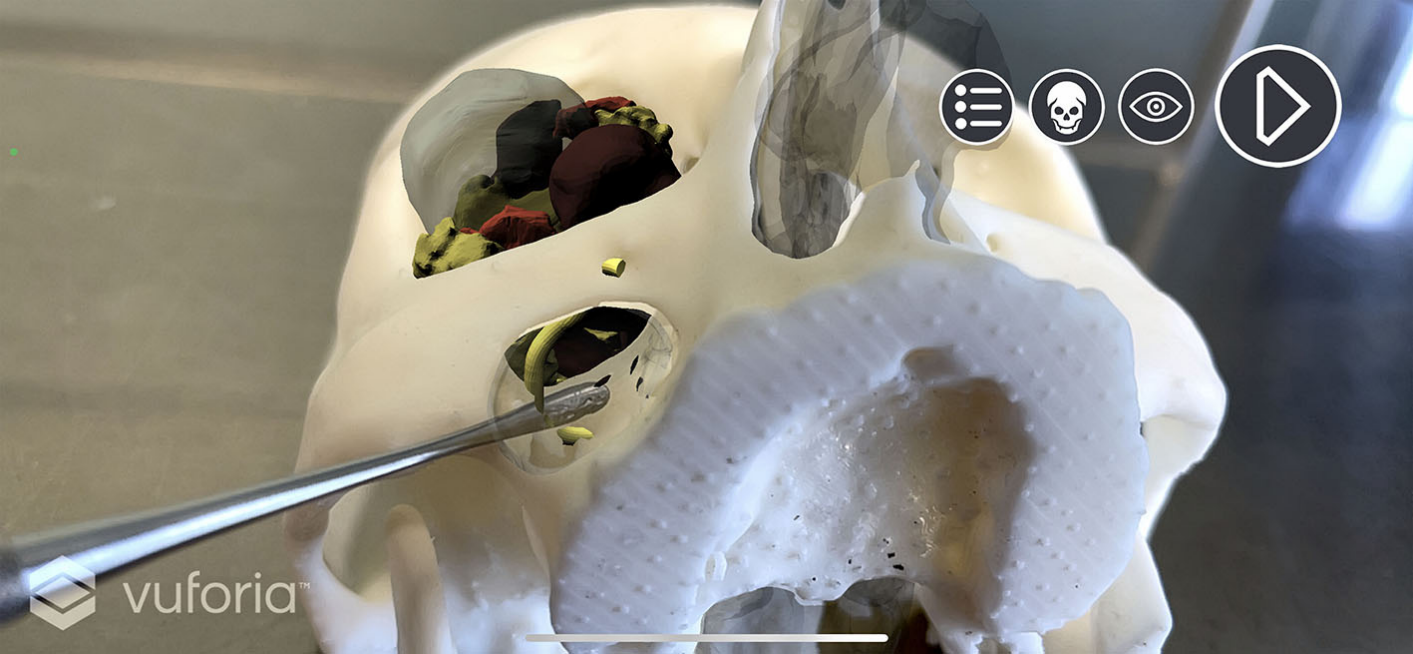
“Holographic pre-surgical training.” Pre-operative simulation with computer-generated visual information. Surgical instruments can be used to simulate interaction with the virtual model of soft tissues during all phases of the simulated virtual endoscopic procedure.

In all cases, pre-operative simulation using AR was used as a guidance system to visualize soft tissue and the process of tumor excision while the surgeon handled the physical 3D printed model and moved surgical instruments (Figure 9).

## Discussion

Compared with the past, modern surgeons consistently amplified their armamentarium, which now includes technological applications at all levels, ranging from image-guided surgery to rapid prototyping, customization of devices, and advanced visualization. We have simplified four areas that make up the technological backbone of maxillofacial surgery: virtual surgical planning, 3D printing, navigation, and AR. Two additional areas, namely, piezosurgery and ultra-high-definition endoscopy, represent the natural evolution of bone cutting and endoscopy, and have today undergone consistent improvements.

We chose the transantral approach for the enucleation of orbital masses arising in the deep inferior orbital space as the ideal setting to present how a multilevel application of technology can not only improve the surgical technique, but also the whole pre-operative phase focused on the study of the single case.

At present, the literature provides little evidence on the use of the transmaxillary endoscopic approach to excise masses located in the inferior orbital compartment. Although the transmaxillary portal anatomically represents the safest and most direct path to reach the orbital floor (10), orbital masses of the inferior orbit are generally excised using traditional transcutaneous approaches, which do not take advantage of endoscopic vision and bear an intrinsic risk of unfavorable scarring (11).

Anatomically, the transantral approach provides the most-straight trajectory to the inferior orbital space through the orbital floor. Our planning approach consists in the preliminary definition of two interrelated elements, namely, the transmaxillary portal, which identifies the opening on the anterior wall of the maxillary sinus, and the transorbital portal, represented by the osteotomy on the orbital floor, which provides access into the intraorbital compartment. Our strategy is to design such portals with the same size, allowing to use the anterior maxillary wall to reconstruct the orbital floor in the eventuality that the original orbital floor breaks during the osteotomy phase, a method that our group has already described for the endoscopic transmaxillary treatment of internal orbital floor fractures (12). Compared with traditional transcutaneous incisions, this approach provides several advantages, including the absence of visible scars, an almost direct vision of the orbital floor up to the orbital apex, which can be further improved using a 30° optics, and, as mentioned, it makes autogenous bone available for orbital floor reconstruction, if needed.

Moreover, in contrast with traditional nasal endoscopy, the maxillary sinus offers an empty and safe space, where no crucial structures are present, allowing to freely maneuver surgical instruments and the optics to scope the anatomy from every desired angulation (9, 13). Previous reports on the transantral endoscopic approach only describe its adoption in the setting of orbital decompression (14–16). On the other hand, excision of masses arising in depth in the orbit has only been reported in conjunction with transnasal endoscopic approaches (1, 17–19), including masses of the inferior orbit, which are well-documented in the work of Arai et al. (1). Some potential advantages of transnasal endoscopy should also be considered before planning the procedure, including functional preservation of ciliary mucosal cleaning, less infraorbital swelling, less infraorbital sensory disturbance, and avoidance of the anterior maxillary wall osteotomy.

In our opinion, the use of nasal endoscopy can be avoided for masses just above the orbital floor or with mild extension to the medial wall, which inferiorly is contiguous with the orbital floor. Nasal endoscopy does not provide a linear access to the medial orbital wall and often requires to cause additional iatrogenic damage to facilitate the passage of instruments in a very narrow space, including turbinate luxation, uncinectomy, and bullectomy, which contribute to substantial bleeding and impaired vision during surgery. On the contrary, the transantral endoscopic access provides a wide space to maneuver surgical instruments and represents a direct access to the inferior orbit, which is entirely exposed once the orbital floor is removed. To minimize complications of this approach, care has to be taken when dismantling the orbital floor not to injure the intraorbital nerve, which can be immediately visualized just beneath the mucosal layer, and whose position is pre-determined by virtual surgical planning and virtual endoscopy. As suggested by Donofrio et al. (9), the infraorbital canal subdivides the orbital floor into two halves, which can be separately opened depending on whether the orbital mass is dislocated more medially or laterally. When the mass size requires removing the whole orbital floor, the infraorbital nerve is gently freed from its canal and displaced during surgical maneuvers. However, the transantral endoscopic approach is not devoid of complications, which might include accidental damage to the nearby maxillary and sphenopalatine artery, disruption of the natural drainage in the maxillary sinus that may require future surgery, as well as closure of the maxillary wall osteotomy. In rare circumstances, even ophthalmological complications might occur; therefore, the presence of an ophthalmologist within the surgical team would represent an additional improvement to provide intraoperative monitoring of the pupil and to manage the eventuality of an orbital hemorrhage during the dissection or at the end of the procedure, as well as its consequences in the postoperative period.

Virtual reality and computer-generated geometrical models today can trustfully reproduce anatomy and disease processes and are therefore indispensable for the pre-operative study of the patient and planning of surgical approaches. For instance, the choice of which area of the orbital floor should be excised in relation to the infraorbital canal is made highly intuitive by assigning the orbital floor a semitransparent shader and highlighting the orbital mass above. Virtual planning also plays a crucial role to study anatomical relationships between the pathological process and surrounding structures, including extraocular muscles and the optic nerve, not even to mention its well-consolidated utility in planning correctly sized osteotomies. The conjunction between virtual surgical planning and surgery is undoubtedly represented by intraoperative navigation, a well-consolidated technology, whose use, however, has never been reported in endoscopic transantral approaches (3). In particular, the wide opening provided by the maxillary portal enables surgeons to navigate through the antral wall within the maxillary sinus, up to the inferior orbit, allowing for instance to be assisted by virtual planning in designing the osteotomy for the orbital floor, and to promptly identify anatomical structures as dissection proceeds.

In this paper, we introduce the concept of “animated virtual surgical planning,” namely, the possibility to simulate movements in the setting of virtual surgical planning, instead of designing purely static entities (4). This step requires proficiency with animation software, and the user can apply a variety of rigid body transformations and deformers to reproduce the variation in time of an object position as well as its dynamic modifications in shape, as shown by Supplementary Video 1. It is a highly innovative concept in the field of computerized planning for surgery, where the most widespread application of virtual planning is limited to osteotomy design. Applicating the animated VSP in the context of the transantral approach for orbital lesions translates in the possibility to simulate the excision of the tumor, which is useful to understand which path is the most comfortable to remove the lesion and therefore, how surgical accesses should be designed. To adapt animated VSP to an endoscopic procedure, we designed a virtual replica of the endoscope based on its effective size, linked to a virtual camera allowing to capture the image as if the surgeon were seeing through the real optics. The variation in time of the endoscope position, based on the optimal image simulated for each surgical phase, was then merged with the animation of the surgical procedure, allowing to simulate an entirely endoscopic vision for each surgical phase. In our opinion, this result is of great interest because of its potential advantages in simulating endoscopic procedures, and it could answer potential questions, including the choice of the most appropriate optics for each phase, which structures the surgeon will encounter before reaching the target and anatomical relationship between lesions and tissues scoped from an endoscopic vision. This differs from any other application of virtual endoscopy, consisting generally of a fly-through vision throughout a rendered CT model with no use of virtual models, and, most of all, the absence of animations (20–22).

The subsequent step toward further evolution in the pre-operative study of the patient concerns the adoption of AR, a technology destined to revolutionize image-guided surgery, but which still suffers from many inaccuracy biases and thus still very limited in its clinical application (8, 23–25). To make a step forward, we applied AR in the pre-operative study of the case, using a 3D printed phantom of the skull of the patient, merged with the animated planning exported from the animation software. Compared with virtual endoscopy, which still provides a two-dimension image, AR animated planning brings the pre-surgical training in a multidimensional scenario, allowing to handle a real model overlapped with the animated planning. This “holographic pre-surgical training” also offers the surgeon or learner the possibility to physically interact with the virtual image using real surgical tools, such as scalpels, elevators, and optics, within the boundaries provided by the physical 3D printed model, combined with the virtualization of surgery which is accurately overlapped on the real-world replica.

However, it should be acknowledged that this workflow requires both hardware and software availability, which not all centers might afford, as well as competencies in translational research, which are not routinely provided in traditional medical education. Although the presented workflow has the advantage of describing an AR application using commonly available technology, consisting of a high-level smartphone, intraoperative guidance requires a surgical navigator, which might not be present in all centers. However, software plays a prominent role in this protocol, and we are aware that medically certified software licenses have a considerable impact on the hospital budget, although for both Maya and Unity free education licenses are available for universities. Moreover, a substantial investment, both in terms of time and effort, has to be considered for clinicians wishing to implement this technology in their centers to acquire additional knowledge in medical image segmentation, animation techniques, and programming. This approach is very preliminary in its conceptualization, but, in our opinion, it may inspire subsequent development which may lead to surgery simulators based on AR, providing surgeons the possibility to anticipatively visualize all the surgical steps before the real surgery is performed. In a future perspective, further studies including more patients might investigate the advantages provided by the adoption of this technological workflow for the transantral approach in relation with conventional techniques.

## Conclusions

This work aims to conceptualize the mindful use of technology in a multilevel approach, which considers several different applications for the pre-operative study of the patient, individualized training, and surgery. The transantral approach is one of the most prominent examples in our experience that can benefit from a complete implementation of technology, yielding a minimally invasive method to excise orbital masses of the inferior compartment with decreased morbidity and excellent simulation capabilities. We advocate that new generations of surgeons should master technology to maximize their benefit in the pre-surgical study of the case and the accuracy and reduced invasiveness of the procedure.

## Data Availability Statement

The original contributions presented in the study are included in the article/Supplementary Material, further inquiries can be directed to the corresponding author.

## Ethics Statement

The studies involving human participants were reviewed and approved by Institutional Review Board, University of Udine, Protocol Number: IRB_45_2020. The patients/participants provided their written informed consent to participate in this study.

## Author Contributions

AT designed the study, performed virtual endoscopy, created virtual models, 3D printed models, and wrote the full paper. LA designed augmented reality interface and contributed to the manuscript. SS and FC supervised the manuscript. RN contributed to the manuscript. MR coordinated the research team and approved the final manuscript before submission. All authors contributed to the article and approved the submitted version.

## Conflict of Interest

The authors declare that the research was conducted in the absence of any commercial or financial relationships that could be construed as a potential conflict of interest.

## Publisher's Note

All claims expressed in this article are solely those of the authors and do not necessarily represent those of their affiliated organizations, or those of the publisher, the editors and the reviewers. Any product that may be evaluated in this article, or claim that may be made by its manufacturer, is not guaranteed or endorsed by the publisher.

## Supplementary Material

Supplementary Video 1 Final rendering of virtual endoscopy and animated surgical planning.

Supplementary Video 2 Augmented reality for orbital surgery in a pre-clinical phase. 3D registration of virtual and real objects is shown through the alignment of the 2D GuideView Image and the corresponding anatomical traits. Simulated endoscopic transantral route is shown from the inferior view, and sinuses are hidden to show the orbital floor seen through the transmaxillary access. Depth mask is applied, the skull model is hidden, and the sinuses are rendered in transparent mode to correctly analyze how the soft tissues are visualized upon the real model. Lastly, the animated augmented reality surgical planning is played, and the tumor is correctly seen while moving across the extraction route.

Supplementary Video 3 Key steps of the endoscopic surgical procedure.
